# Evidence of a Large-Scale Functional Organization of Mammalian Chromosomes 

**DOI:** 10.1371/journal.pgen.0010033

**Published:** 2005-09-09

**Authors:** Petko M Petkov, Joel H Graber, Gary A Churchill, Keith DiPetrillo, Benjamin L King, Kenneth Paigen

**Affiliations:** The Jackson Laboratory, Bar Harbor, Maine, United States of America; Cornell University, United States of America

## Abstract

Evidence from inbred strains of mice indicates that a quarter or more of the mammalian genome consists of chromosome regions containing clusters of functionally related genes. The intense selection pressures during inbreeding favor the coinheritance of optimal sets of alleles among these genetically linked, functionally related genes, resulting in extensive domains of linkage disequilibrium (LD) among a set of 60 genetically diverse inbred strains. Recombination that disrupts the preferred combinations of alleles reduces the ability of offspring to survive further inbreeding. LD is also seen between markers on separate chromosomes, forming networks with scale-free architecture. Combining LD data with pathway and genome annotation databases, we have been able to identify the biological functions underlying several domains and networks. Given the strong conservation of gene order among mammals, the domains and networks we find in mice probably characterize all mammals, including humans.

## Synopsis

The arrangement of genes along chromosomes affects their function as well as the likelihood that particular combinations of genes will be inherited together, and evolution has had many millions of years to optimize these arrangements. Because the arrangements are nearly identical in all mammals, one can use the powerful techniques of mouse genetics to explore their roles in our own genomes. The authors find that genes that cooperate in bringing about various cellular and physiological functions, such as immune responses, are often clustered together on chromosomes, and that detailed maps of these relationships can be built. The new techniques have proven so powerful that they can identify functional interactions among genes that are not even on the same chromosome. Beyond illuminating the evolutionary pressures that brought them about, mapping these arrangements will be of great utility in the ongoing searches in many laboratories for the genes underlying our common diseases, such as cancer, heart disease, and diabetes.

## Introduction

The physical and functional organizations of eukaryotic chromosomes are correlated outcomes of evolution potentially reflecting interactions among structural, regulatory, and functional factors. As typified by the α and β globin gene clusters, tandem duplications can give rise to gene families whose members develop divergent, but still related, functions over time. Gene clusters may arise as a means of promoting their coregulation through regional controls of chromatin structure and expression, and there is now considerable evidence, well summarized by Hurst et al. [1], that for variety of eukaryotes, including yeast, *Caenorhabditis, Drosophila,* higher plants, and mammals, genes sharing expression patterns are more likely to be in proximity than would be expected by chance. And finally, Fisher [2] and later Nei [3,4] have argued on theoretical grounds that when genes interact epistatically, evolutionary selection will promote their genetic linkage as a means of enhancing the coinheritance of favorable allelic combinations. Dobzhansky and others have provided experimental evidence on the importance of coadapted sets of alleles in their studies analyzing the fitness of chromosomal inversions in *Drosophila* [5]. Although there is limited molecular evidence in this regard, we can presuppose that allelic coadaptation is most likely to be effective when the various gene products participate in the same biological function. In yeast, proteins within the same macromolecular complex are about twice as likely to be encoded by genes on the same chromosome as would be expected by chance [6], and for a variety of eukaryotes, including humans, the genes encoding the enzymes of some pathways of intermediary metabolism occur in multiple clusters [7]. Additionally, several very specific, albeit isolated, examples of functional clustering are known in mammals, including the major histocompatibility complex, reviewed in [8], and the four HOX clusters [9].

Obviously, structural, regulatory, and functional factors are not mutually exclusive possibilities underlying selection, and while they could act in concert, that is not a requirement. Genes coexpressed in the same tissue may participate in distinct functions, the multiple functions of hepatocytes being a prime example, and a particular biological function may involve interaction among multiple cell types, as occurs in the mammalian immune response.

To address the question of functional clustering further, we have turned to inbred mice, which provide a unique and readily accessible experiment on evolutionary selection. Nearly a million years ago the species *Mus musculus* diverged into three geographically distinct subspecies, *M. m. domesticus, M. m. musculus,* and *M. m. castaneus,* along with a fourth subspecies, *M. m. molossinus,* that is an ancient fusion of the latter two subspecies. Over the last few centuries amateur mouse fanciers intercrossed these subspecies, and beginning less than a hundred years ago these genomic mixtures were used as the source of many of our present laboratory inbred strains. The process of inbreeding to homozygosity imposes intense selective pressures; all efforts among some species have failed, and with mice, only a fraction of initial attempts succeeded. Accordingly, we can expect that if clustering of functionally related genes is a common feature of mammalian genomes, there is likely to be selection for coadapted allelic combinations among the genes encoding functions that influence fitness and survival during inbreeding. This would result in regions of linkage disequilibrium (LD) among inbred strain genomes; i.e., some allelic combinations should occur more often than expected by chance.

This prediction can be tested using the large numbers of single-nucleotide polymorphism (SNP) genomic markers that have been typed on multiple mouse strains. In doing so, we found a substantial fraction of the mouse genome present in LD domains averaging several megabases in size, along with experimental evidence that the preferred configurations of these domains confer a considerable selective advantage during inbreeding. Moreover, the LD data show that domains identified in this way interact in complex networks across the genome. Correlating domain and network maps with information from pathway and Gene Ontology (GO) databases has made it possible to identify some of the underlying functions on which selection has acted.

## Results

### Strains and Single-Nucleotide Polymorphisms

The starting point for this analysis was a dataset describing the distribution of alleles at 1,456 SNPs, chosen for their high information content, among a set of 60 common and wild-derived inbred mouse strains chosen for their genetic diversity. They represent all of the SNPs and strains meeting a set of minimum requirements within a larger set (1,638 SNPs, 102 strains) originally developed as a mouse mapping panel [10]. The final dataset was characterized by a median minor allele frequency of 0.32 (minimum 0.1) to provide statistical robustness; a median frequency of allelic differences among pairs of strains of 42.8% (minimum 20%) to remove “sibling” strains that would otherwise introduce artifactual LD; and a median frequency of successful SNP determination of 98.3% (minimum 90%) to avoid possible biases from failed typings. The identity of these strains and the phylogenetic relationships among them are indicated in Figure 1, which was constructed using neighbor-joining methodology [11].

**Figure 1:**
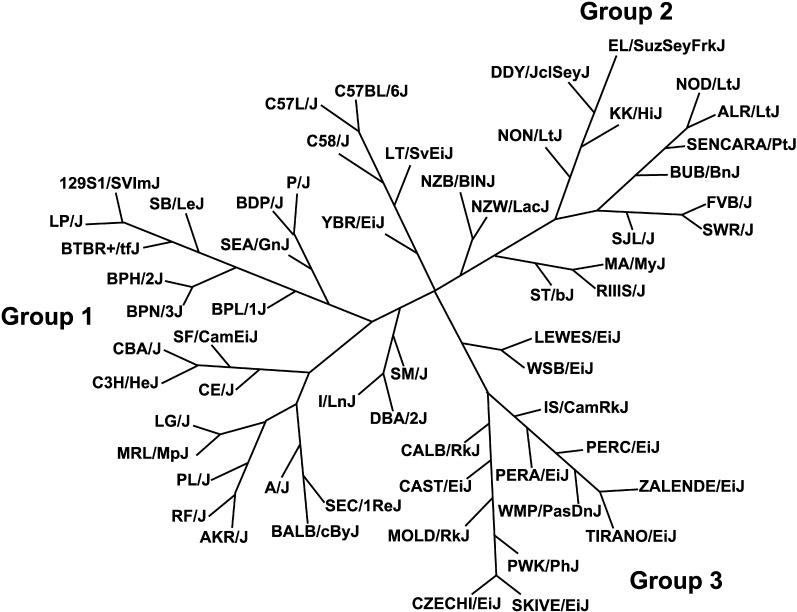
Neighbor-Joining Distance Tree of the Mouse Strains Used in This Study The length and angles of the branches have been optimized for printing and do not represent actual phylogenetic distances. Group 1, Bagg albino, 129, and DBA-related strains; group 2, Swiss mice and Asian strains; group 3, wild-derived strains.

### Estimating LD

Historically, the term linkage disequilibrium has been applied to populations, referring to the nonrandom association of alleles at linked genes; i.e., over time, recombination has failed to establish a random assortment of alleles, some combinations occurring more often than expected by chance. Although in mammalian genetics LD has been traditionally applied to linked genes, we are reluctant to introduce new terminology, and so have extended the definition to any pair of markers, regardless of location. Following the discussions and recommendations of Hedrick [12] and Devlin and Risch [13] on the use of alternative methods, we have estimated LD using *D′,* the difference between the observed frequency of an allelic combination and its random expectation, relative to the maximum deviation possible given the allele frequencies of the two markers [14,15]. *D′* corrects for differences in allele frequencies and describes LD equally well when there is selection for or against the combination of majority alleles. A cumulative Fisher's exact test (FET) was used to compute the probability *(p_FET_)* of obtaining an equally or more extreme distribution under the null hypothesis of random allelic association between pairs of SNPs. This approach has the advantage of providing separate estimates of the extent of disequilibrium and the likelihood of its being due to chance, and is especially valuable for markers with lower minor allele frequencies [12,16,17]. In addition to *D′,* we considered *X* ^2^, *r* ^2^ and mutual information measures of disequilibrium on the same data. All of these measures were highly correlated, above 0.9, and here we report our results in terms of *D′*.

### LD Patterns Reveal the Presence of Domains

The observed data for the 60 strains (Figure 2, solid squares) shows that among marker pairs less than 1 Mb apart, a considerable excess—approximately 44%—are in disequilibrium at *p_FET_* < 0.001, and that disequilibrium decays slowly as the distance between pairs increases, reaching a lower bound of approximately 1% at distances above 20 Mb. We estimated the false discovery rate at *p_FET_* < 0.001 to be 0.09 via the conservative method of Benjamini and Hochberg [18]. Tests with two randomized datasets indicate that these observations of high LD are highly significant and not a consequence of either marker location or allele frequency distributions. In one set, marker locations were randomized while maintaining the assignments of alleles to strains (Figure 2, red triangles), and in the other set the assignments of alleles to strains were randomized while preserving allele ratios and marker locations (Figure 2, solid circles). Neither control set indicated dependence on marker separation, with a uniform 1% of the pairs in LD for set 1, a value similar to that observed in interchromosomal marker pairs (unpublished data), and less than 0.1% of the pairs in LD for set 2, approximately conforming with random expectation.

**Figure 2:**
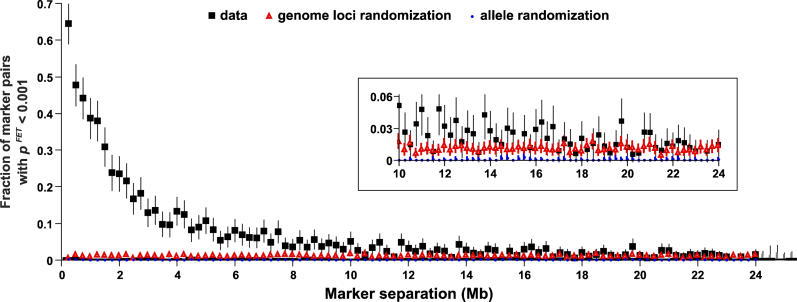
Dependence of the Fraction of Markers in LD from the Distance between Them Fisher's exact test was used, *p_FET_* < 0.001 unadjusted. Solid squares, actual data; red triangles, randomized genomic positions of the markers; solid circles, randomized alleles and strains.

Almost certainly, these calculations underestimate the true extent of LD, as the requirement of *p_FET_* < 0.001, chosen to keep the false discovery rate low, also increases the false negative rate.

Figure 3 displays the interactions between all pairs of markers on Chromosome 14, a chromosome exhibiting extensive LD. Marker coordinates on the chromosome, expressed in Mb, form the two axes; where the coordinates for a pair of markers intersect, *D′* and the base-ten logarithm of 1/*p_FET_* are plotted above and below the diagonal, respectively. Points along the diagonal represent closely spaced markers. We used a dynamic-programming method (Materials and Methods) to identify the most probable domains of LD, which appear as blocks along the diagonal. Depending on the exact parameters chosen for dynamic programming, the mouse genome contains several hundred LD domains, occupying one-fourth to one-third of the total length.

**Figure 3:**
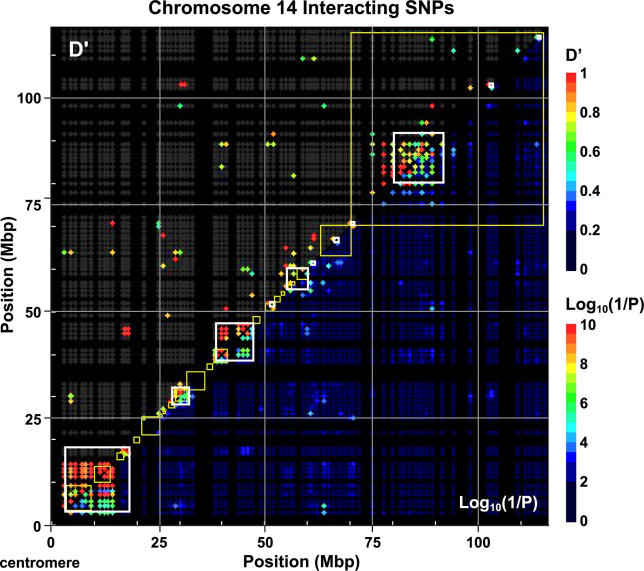
A Representation of LD between Marker Pairs on Mouse Chromosome 14 Reveals a Domain Structure LD is plotted as *D′* and log_10_ (1/*p_FET_*) above and below the diagonal, respectively. The x- and y-coordinates are NCBI Build 33 genome positions for SNPs. Black regions reflect genomic sequence not covered in this SNP set (i.e., missing data). To highlight pairs of interest, *D'* values have been suppressed (plotted as gray) for marker pairs with *p_FET_* > 10^−3^. White boxes represent LD domains, identified as described in the text. Yellow boxes represent regions of synteny identified through mouse-rat-human-chicken comparison [19].

At the present level of resolution, domains defined in this manner appear related to the evolutionarily conserved syntenic blocks previously identified in a joint analysis of the mouse, rat, human, and chicken genomes [19]. It will be of considerable interest to see if this relationship persists when more extensive data become available.

To confirm that these domain maps are independent of our SNP panel, Chromosome 14 was tested using two other panels with denser SNP coverage, albeit on fewer strains. These panels were generously provided by Tim Wiltshire at GNF-Novartis [20] and Eric Schadt at Rosetta-Merck. The results are almost identical to the patterns in Figure 3 (unpublished data). Although there is no reason to believe it will vitiate our results, we should note as a caution that all presently available mouse SNP panels are limited in their representation of mouse genome diversity, in that they are derived by comparing the genomic sequences of a small number of strains.

### Domains Reflect Inbreeding Selection

Recombinant inbred (RI) lines of mice provide a direct means of testing whether domains result from inbreeding selection. We can ask whether LD domain regions whose allelic composition has been scrambled by recombination have a reduced ability to survive further inbreeding, using as the control regions of the genome that do not show LD. RI lines are nearly ideal for this approach, as they are created by crossing two genetically defined progenitor, inbred strains of mice, obtaining an F2 population in which all allele ratios are 50:50, and then inbreeding a set of new lines from pairs of F2 mice. The result is a new set of inbred mouse strains created from genetically defined progenitors. Within LD domains, if inbreeding during RI line formation favors the survival of preexisting allelic combinations over new ones, the number of lines showing recombination across these regions should be fewer than expected. This effect should be absent from the nondomain regions. The number of RI lines expected to show recombination across a region if there is no adverse selection can be calculated from the rates of recombination seen among the F2 progeny obtained from crosses of the two parental strains. This is provided by the classic Haldane-Waddington equations, in which the fraction of lines recombinant between two autosomal markers is given by *4c/*(*1 + 6c*), where *c* is the recombination fraction in a single generation. This equation, which was originally derived theoretically, has recently been validated by computer simulation [21].

Rates of recombination in crosses between C57BL/6J and either A/J or DBA/2J mice were measured among F2 progeny and then, using the same sets of markers, across the large BXA and BXD sets of RI lines created from these progenitor strains. Domain and nondomain regions were chosen for comparison solely on the basis of the availability of flanking markers and without prior knowledge of recombination frequencies. Comparing a set of domains totaling 108.0 Mb with the set of nondomains totaling 83.8 Mb, we found nearly equivalent single generation recombination rates, both very similar to the overall genome wide recombination rate of 0.55 cM/Mb (Table 1). After subsequent inbreeding, there were markedly fewer than expected RI lines containing recombinant LD domains, but this was not the case for the nondomain regions (*p* = 0.0000003 and 0.57, respectively). We conclude that LD domains are not deficient in normal recombination, and that selection against less favorable allelic combinations is a strong factor generating LD. These results from RI lines are noteworthy in that, unlike the data from inbred strains, there are no issues reflecting common origins, biases of marker selection among multiple strains, or possible effects of allele frequencies, as all input allele ratios are 50:50.

**Table 1:**
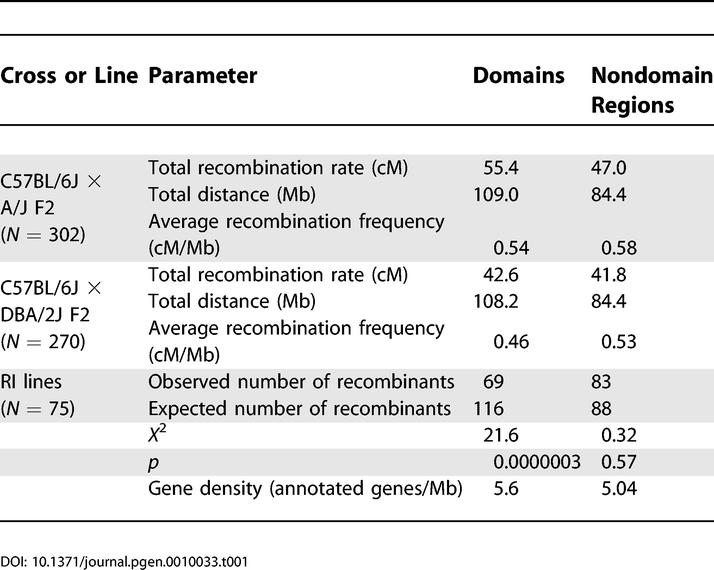
Comparison of Recombination in F2 Crosses and Recombinant Inbred Lines

Confirmation that selection reducing the survival of recombinants occurs on a broad scale during RI line inbreeding was provided by genomewide measures of recombination among sets of RI lines. A comprehensive dataset of 1,575 markers used to describe recombination among 109 RI lines bred from various parental combinations has been assembled, expanded and quality controlled by Williams et al. [22]. The expected apparent genome length among these strains can be calculated using the Haldane-Waddington equations by multiplying the autosome lengths by 4, the X chromosome by 8/3 [21], and reducing the expected length by 1/1.075 (the Williams et al. correction for the average distance between markers). If there is no selection reducing survival of recombinants, the single generation genome length of 1,465 cM should generate an apparent genome length of 5,362 cM. For the 109 strains, this predicts 5,845 recombination events, however, only 4,786 were observed, a deficiency of 18.1% (*X^2^* = 192, *p* < 10^−47^). Every autosome was deficient in recombination, and the deficiency was particularly marked for the X chromosome, 32%. In further evidence of selective forces during inbreeding, the authors noted multiple regions of residual heterozygosity persisting long after they would be expected to be lost by chance.

If the 40% reduction in recombinant survival seen among the LD domains described in Table 1 is typical of LD domains in general, an 18.1% genome wide lack of recombinant survival among RI lines suggests that a substantial fraction of the mouse genome, as much as one-third to one-half, may lie within the LD domains defined by selection during inbreeding. This would agree with our probable underestimate of the true extent of LD in Figure 2.

### Domains Are Not the Result of Deficient Recombination, Gene Content, or Strain Origins

Although reduced recombination is a commonly accepted mechanism generating LD in human populations, as Table 1 shows, recombination frequencies per Mb were not significantly different between LD domain and nondomain regions, or from the genome-wide average of 0.55 cM/Mb. Reduced recombination across LD domains was seen only as a consequence of later inbreeding selection, and can best be described as “lack of recombinant survival” rather than “lack of recombination.”

LD domains are also not regions of particularly high or low gene density; domains and nondomains being quite similar in this regard (Table 1).

Although many of the older inbred mouse strains had their origins in limited populations of domesticated mice, this factor is not sufficient to explain the LD observations. The 60 mouse strains tested (excluding the branch anchored by C57BL/6J which provided the reference sequence for SNP discovery) separate into three phylogenetically distinct groups that conform well to our historical knowledge of their origins (see Figure 1) in which groups 1 and 2 consist of strains derived from domesticated mice and group 3 contains wild-derived strains. If LD is due to commonality of origin, the three groups should behave independently. However, when compared using a rank-order test (see Materials and Methods), there was an appreciable excess over chance of marker pairs showing LD in more than one group (Figure 4). This was also true when any two of the three groups were compared pairwise (unpublished data). These results do not mean that common origin effects are entirely absent from inbred mouse strains, only that selection appears to be a significant mechanism generating LD.

**Figure 4:**
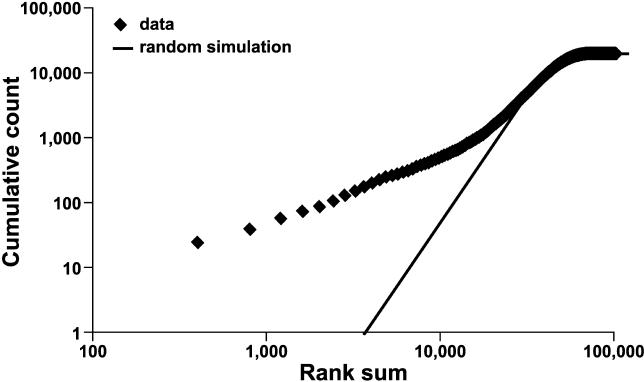
Distribution of Sum of Rank Scores for Marker Pairs Distribution is shown for the three groups of mouse strains as described in Materials and Methods. Deviation from the random simulation indicates sharing of LD pairs between groups.

### Functional Networks Are Contained and Organized within Domains

To investigate the hypothesis that LD domains contain biologically related genes with coadapted alleles, genes from the ten most significant domains (based on dynamic programming) were analyzed for an excess of functionally related genes using the VLAD program (http://proto.informatics.jax.org/prototypes/vlad) that relies on GO [23] annotations. Membership in gene pathways and networks was tested using Ingenuity's Pathways Analysis software (http://www.ingenuity.com), which relies on literature annotation of physical, metabolic, and regulatory interactions between gene products, and, importantly for this purpose, ignores information on gene locations. Genes with common GO annotations were found in five of the ten domains. When analyzed for their pathway content, in addition to a *Hox* cluster on Chromosome 6, four of these domains contained a total of 13 additional Ingenuity pathways, each containing eight to 21 colocalized genes. Collectively, 35% (157/455) of the genes in these pathways were located in single domains.

The densest clustering occurred in the Chromosome 1 domain between 167.2 and 174.2 Mb, a region containing a total of 119 predicted genes (via the ENSEMBL Nucleotide Sequence Database [http://www.ebi.ac.uk/embl]). When all 119 genes were tested for functional relationships, 21 genes proved to be components of an Ingenuity pathway of 35 genes containing two distinct subnetworks linked by the gene *Crp* (Figure 5). The lymphocyte subnetwork spans from *Crp* to *Eat2* and includes genes coding for 15 proteins, among them five Fc receptors and the cell surface antigens Cd48, Cd244, and Ly9. The general functions of the genes in this network include immune response, inflammation, inflammatory disease, phagocytosis, movement of lymphatic system cells, activation and proliferation of leukocytes, and costimulation of T lymphocytes. The *Myc* subnetwork contains ten genes acted on by *Myc* and three genes whose products act on *Myc*. The general functions of this subnetwork include apoptosis (including apoptosis of lymphatic system cells), proliferation of tumor cell lines, transformation of cells, inactivation of mast cells, and cardiovascular system development and function.

**Figure 5:**
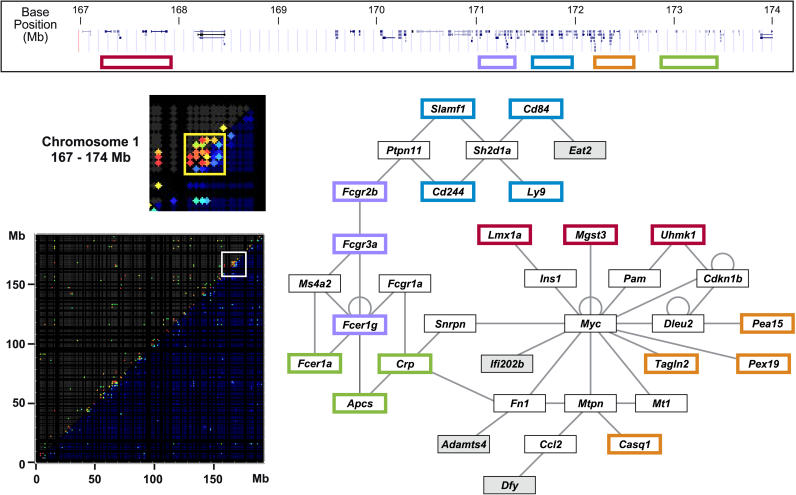
An Example of a Gene Network that Is Largely Contained within an LD Domain Located between 167.2 and 174.2 Mb on Mouse Chromosome 1 Highlight colors on the network plot correspond with the regions shown on the genomic map. The genes in grey boxes are positioned in the same LD domain but not clustered. In this example, eight of the 11 genes in the lymphocyte subnetwork that are in the block *(Eat2, Fcgr2b, Fcgr3a, Fcer1g, Cd244, Ly9, Slamf1,* and *Cd84)* map to within 1.4 Mb. One gene, *Adamts4,* is located within this 1.4 Mb region, but is part of the *Myc* subnetwork. Three additional genes, *Crp, Apcs,* and *Fcer1a,* are within 600 kb of one another and 900 kb away from the other group of eight genes. One set of four genes, *Cd244, Cd84, Ly9,* and *Slamf1,* each bind *Sh2d1a* [44] and are organized sequentially along the chromosome. In humans, missense mutations in *SH2D1A* are associated with X-linked lymphoproliferative disease (XLP) [44], and homozygous targeted mutations of *Sh2d1a* in mice yield immune system abnormalities [45]. Another set of three genes, *Fcgr2b, Fcgr3a,* and *Fcer1g,* that are organized sequentially are Fc receptor subunits. A second set of three genes, *Crp, Apcs,* and *Fcer1a*, are also sequentially organized and directly bind with *Fcer1g* in the case of *Fcer1a* and *Apcs* [46] or bind *Fcgr1a* and *Apcs* in the case of *Crp* [47]. In the *Myc* subnetwork, two groups of genes are located in close proximity to one another. *Mgst3* and *Lmx1a* are within 300 kb of one another. Myc increases expression of *Mgst3* [26], and LMX1A regulates *Ins1* [48], which has its expression downregulated by Myc [49]. *Pex19, Pea15, Casq1,* and *Tagln2* are located within 400 kb. Myc decreases the expression of *Pex19* [26] and *Tagln2* [50]. Mtpn increases the expression of *Casq1* and *Myc* [51]. Myc decreases the expression of *Akt1* [26], and Akt1 increases serine phosphorylation of Pea15 [52].

The probability of finding a pathway with 21 of 35 genes clustered within a contiguous block of 119 genes is very small (*p* = 10^−25^, based on a hypergeometric calculation) even after correcting for the presence of six likely gene duplications, and after Bonferroni correction for the number of contiguous blocks of 119 genes in the genome. The chance of finding 13 such pathways with at least eight clustered genes spread across five LD blocks is even smaller.

The 21 pathway genes colocalized on Chromosome 1 are not all coregulated within a single tissue, indicating that this clustering reflects functional interactions among multiple cell types. Using the Mouse Gene Atlas database of gene expression patterns [24], many of the genes in the immune function branch of the pathway showed broad expression, albeit with an emphasis on expression in bone marrow and lymphocytes. However, two genes in the pathway, *Apcs* and *Crp,* are liver specific in expression, coding for secreted peptides that react with Fc receptors (other members of the pathway) on lymphocytes in the acute phase inflammatory response.

The physical locations of the pathway genes along the chromosome are correlated with the topology of the functional pathway (Figure 5); neighboring pathway genes are clustered in chromosomal subdomains, providing strong support for assembly of LD domains as a result of functional interactions. The two genes sharing liver specific expression, *Apcs* and *Crp,* are located within 200 Kb of each other, further suggesting that a small regulatory domain may be nested within a considerably larger functional domain.

One of the two remaining pathways represented in this domain is connected to the immune function pathway through the gene *CD244;* the joined pathways contain a total of 69 genes, of which 31 are located within the Chromosome 1 domain.

Four noncontiguous markers in the Chromosome 1 domain are also in LD with a single marker on Chromosome 6 that is located near the *Cdkn1b, Bcl2l14, Emp1,* and *Csda* genes, which are involved in apoptosis, cell cycle arrest, and cell growth. Myc decreases the expression of *Cdkn1b* and increases the expression of *Emp1* [25] and *Csda* [26], and although there is no evidence that *Bcl2l14* is regulated by Myc, Bcl2 protein family members are known critical regulators of apoptosis [27]. The LD observed between functionally related genes on Chromosomes 1 and 6 provides additional evidence for selection of coadaptive alleles, albeit on different chromosomes.

### Domains and Markers Associate in Scale-Free Networks

The observation of LD between Chromosomes 1 and 6 is typical; domains on one chromosome are often in LD with domains on other chromosomes or with distant domains on the same chromosome, even when the two are separated by domains that are not in LD with either (Figure 6). Figure 6A expands Figure 3 to include Chromosomes 14–17, illustrating the fact that domains can be in disequilibrium with other distant domains or individual markers on the same or different chromosomes, suggesting the existence of interacting networks. Using the LD data, a network graph was constructed in which the markers are nodes, and edges are created between all marker pairs with *D′* > 0.8 and *p_FET_* < 0.001. To identify the most highly connected subnets, the display was restricted to include only nodes that were part of a fully connected subnet (clique) of at least six nodes. As shown, the highlighted nodes in Figure 6B correspond to the same interchromosome networks highlighted in Figure 6A. Relaxing any of these very stringent requirements (*D′, p_FET_,* or connectivity) vastly expands the network.

**Figure 6:**
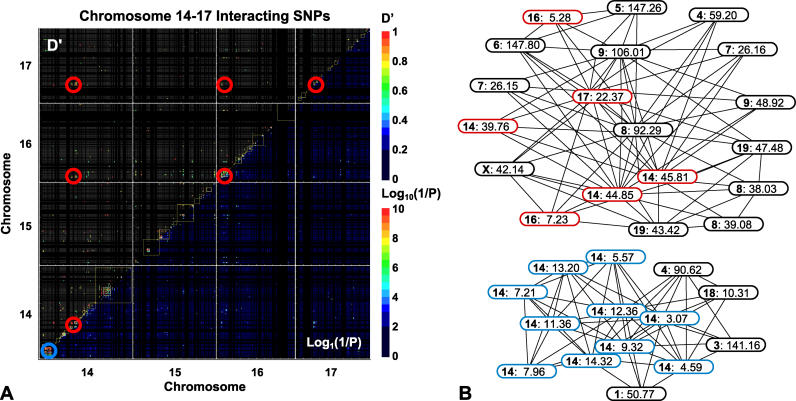
Interchromosomal Plots of LD Reveal the Presence of Putative Interaction Networks (A) A plot of the disequilibrium between pairs of SNP markers on mouse Chromosomes 14–17. Plot parameters are identical to Figure 2. The members of two mutually exclusive, and completely connected, putative interaction networks are highlighted with red and blue circles, chosen to correspond with the highly connected network cores shown in (B). (B) A representation of two reduced, highly connected networks was created by restricting the edges to marker pairs with *D′* ≥ 0.8 and *p_FET_* ≤ 10^−3^. To highlight only the most connected markers (nodes), the graph was reduced to show only nodes that were part of biconnected components (cliques) consisting of six or more nodes, and only components that include markers from Chromosomes 14–17, as shown in (A). Highlighted nodes correspond to the highlighted regions in (A).

Further confirmation of the existence of interchromosomal networks is provided by the RI lines data referred to above [22] which show substantial LD between markers on separate chromosomes, and, importantly, these associations form networks (e.g., chromosomes 8,10,12,13). These results extend earlier observations among RI lines reporting LD between several pairs of markers on separate chromosomes [28].

Metabolic and regulatory networks in lower organisms form scale-free networks [29–32]. In these networks, the frequency of molecules (nodes in network terminology) with *n* connections to other molecules is a negative exponential function of *n;* that is, as *n* increases, there is a constantly declining fraction of nodes with that number of connections. The distant interactions detected by LD might also show scale-free properties when the nodes correspond to genes coding for particular macromolecular gene products (protein or RNA) and possibly DNA binding sites, and the connections correspond to metabolic or physical interactions among these gene products. Scale-free behavior would also demonstrate that the LD networks are nonrandom and hence not a consequence of chance associations. To avoid the complications of local LD, the analysis only considered distant interactions, i.e., those between pairs of markers on separate chromosomes or at least 20 Mb apart on the same chromosome. With this limitation, interactions among the 1,456-marker set tested conformed well to a scale-free network, where *f_C_*(*n*), the fraction of markers with *n* associations, is *f_C_*(*n*) = 0.79*n* ^−1.73^, and this fraction is clearly different from the chance expectation that would be given by a Poisson distribution with the same average number of connections per marker (Figure 7).

**Figure 7:**
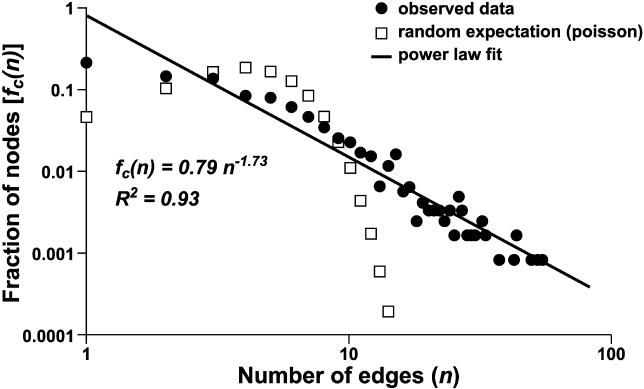
The Connectivity among Pairs of Markers Shows a Scale-Free Character The graph plotting the frequency of markers having *n* connections was created by designating each SNP marker as a potential node in a network, and considering a pair of markers to be connected if *D′* ≥ 0.8 and *p_FET_* ≤ 10^−3^. To eliminate local effects, all pairs of markers separated by less than 20 Mb on a common chromosome were excluded from the analysis. The regression line was calculated for the best fit to the observed data. The deviation from the theoretical straight line at low connectivity is expected for a finite population when the average connectivity is greater than one, and the observed deviation agrees in magnitude with that obtained in computer simulations. The open squares are the results expected for the same average number of connections per marker if the frequency of markers with *n* connections conformed to a random Poisson distribution.

### Biological Functions Correlate with LD Networks

A search for biological functions that might underlie the LD networks was carried out using every term in the GO annotation database [23] that had more than 50 and less than 500 genes assigned to it. Each gene set was tested for excess LD by counting the number of gene pairs in LD when judged by the stringent requirements of *D′* > 0.8 and *p_FET_* < 0.001. SNPs within 2 Mb of each gene (based on their NCBI Build 33 genome coordinates) were used as markers for the gene, and the random expectation determined by carrying out the same analysis on 2,000 sets of an equal number of genes randomly chosen from the panel of annotated genes in ENSEMBL. The results show that after using the conservative Benjamini-Hochberg correction for multiple testing, there are a number of biological functions with an excess of distant genes in LD (Table 2).

**Table 2:**
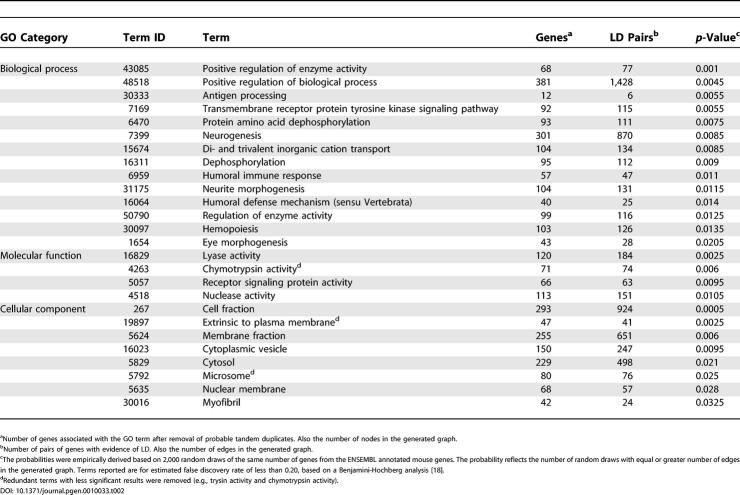
A Summary of Systematic GO Analysis of the LD Network

The validity of this test is supported by prior expectation of a positive result for the term “eye morphogenesis.” It is well known among mouse geneticists that mouse handlers picking mice from a cage inadvertently, but invariably, pick any visually impaired or blind mice first; simply put, the other mice do a better job of running away from the forceps. The result is strong selection for visual impairment.

## Discussion

The relation between LD domains and the “haplotype blocks” [33–38] or “haplotype networks” [38] described in the literature requires clarification. A haplotype is a particular sequence, either of base pairs or allelic markers, in a defined DNA segment. A haplotype block is defined as a contiguous segment of DNA in which the number of observed haplotypes in a population is a small fraction of the total number possible. Such reduced sequence variability will necessarily lead to LD such as we have observed; thus, at their core, LD domains and haplotype blocks reflect the same phenomena.

Various computational approaches have been used to identify and characterize haplotype blocks. These include using a confidence interval restriction on all adjacent marker pairs within the block [37], and using a dynamic programming algorithm based on *D′* values [35,38,39]. Haplotype blocks have also been defined purely by their lack of recombination hotspots or unlinked sequences within the block. Phillips et al. [35] have used extensive simulation to demonstrate that haplotype block structures could arise in the absence of selection simply through extremes of marker density and minor allele frequencies, two pitfalls we have been careful to avoid. Several additional, potential sources of haplotype block structure have been postulated, including (i) heterogeneous recombination, (ii) natural selection, (iii) population bottlenecks, and (iv) population admixtures. Haplotype networks resemble LD domains in that they do not explicitly forbid the inclusion of unlinked markers, a restriction commonly found in neighbor-based definitions [33].

Our statistical definition of LD domains relies on a dynamic programming approach, which explicitly allows contributions from all marker pairs in a putative domain and does not limit the analysis to adjacent or close neighboring pairs of markers (see Materials and Methods). In effect, our method identifies the most probable assignment of domains by maximizing a local sum based on the mutual information for all pairs of markers contained within a defined domain. We have retained the term “LD domain” for our own data for several reasons. First and foremost, our domains are operationally defined by LD, and this is the critical parameter in their definition. Additionally, (i) the literature is not entirely consistent in its definitions of haplotype blocks; (ii) unlike the human case, mouse LD domains do not differ from nondomains in recombination activity or gene content; (iii) the LD domains we observe are an order of magnitude larger than previously reported haplotype blocks in mice and even more so for those reported in the human genome, and (iv) the markers within domains associate across chromosomes in scale-free networks. Finally, and very importantly, the LD domains of mice appear to have a functional basis, arising as a consequence of inbreeding selection for compatible sets of genetically linked, functional elements, an association that, as far as we know, has not yet been made for haplotype blocks.

It is difficult to escape the conclusion that the selective factors acting to generate LD domains and networks during inbreeding reflect clustering and/or interaction of functionally related elements along chromosomes, thereby providing an opportunity for expanding our limited knowledge of the forces that drive molecular evolution in general, and coadaptation of alleles in particular. Chromosome maps and pathway networks are reflections of each other, with the potential of being mutually informative.

These observations are consonant with the theoretical suggestions of Fisher [2] and Nei [3,4] that evolution promotes the development of genetic linkage as a means of enhancing the coinheritance of favorable allelic combinations, and with the experimental work of Dobzhansky and others emphasizing the existence of coadapted gene sets in explaining the population genetics of *Drosophila* [5]. Inbred mice have provided a unique evolutionary experiment confirming these concepts in that they are derived from progenitor populations that had over a million generations of prior evolution in which to develop coadapted sets of alleles within local populations or subspecies. Laboratory matings arbitrarily scrambled these combinations. The resulting progeny were then subjected to intensive selection during inbreeding for the many epistatic interactions among genes, reinforced by the imposition of homozygosity, processes that effectively selected particular allelic combinations.

The LD domain on Chromosome 1 (167–174 Mb), which contains a large functional network, is a microcosm of the multiple factors—structural, regulatory, and functional—that give rise to genomic organization. It includes separate examples of apparent gene duplications, tissue-specific coregulation of adjacent genes, and functional signaling between cell types. Eight of the 21 functionally related genes within this domain might be related by gene duplications. Four, located within a 300-kb interval *(Slamf1, Cd84, Lys,* and *Cd244),* are related members of the immunoglobulin superfamily, and almost certainly arose as gene duplications. The genes *Fcgr2B* and *Fcgr3A* both code for Fc family receptors and are less than 100 Kb apart, suggesting that this pair also likely arose by gene duplication, and two other genes, *Crp* and *Apcs,* which share 50% sequence identity, are less than 200 Kb apart, again suggesting an ancient gene duplication. At the regulatory level, *Crp* and *Apcs* show a liver-specific expression, and may well be coregulated [40]. Thus, the Chromosome 1 domain is likely the outcome of a complex pattern of evolution reflecting structural, regulatory and functional selective factors, all acting to create this functional domain.

Comparative genomics has shown that gene order is a highly conserved feature of mammalian and even more evolutionarily distant chromosomes [19,41]. The selective pressures that originally drove the clustering of functionally related elements on mammalian chromosomes must have acted prior to the first divergence of mammalian orders more than 75 million years ago, and are not unique to any one order of mammals, much less the genus *Mus*. They almost certainly involved functions essential for mammalian existence. We can expect that elucidating these functional connections will be revelatory for all mammals, including ourselves, and suggestive evidence that LD domains resulting from selection might also occur in human populations has been reported recently [35,38,42].

What is notable about our present results is the extent to which functional clustering appears to be present in the mammalian genome. However, what must be emphasized is that while the LD domains of mice have been valuable in leading to this view, their sharply delimited edges probably reflect the particular selection pressures of inbreeding mice in a laboratory environment, rather than evolutionary forces in general. It is likely that functional clustering is as common in the non-domain regions, but less apparent, as it is not under inbreeding selection. Accepting this broader role for evolutionary selection suggests the possibility that all, or nearly all, of the mammalian genome is a linear continuum of functionally related elements and that clusters of functionally related genes may well be interdigitated among each other. Indeed, virtually every search for the many loci underlying complex traits not under laboratory selection, such as disease susceptibility, has revealed multiple epistatic interactions. The functional anatomy of the mammalian genome must be more complex than the fraction we have been able to observe so far.

## Materials and Methods

### Definition of domain boundaries through dynamic programming.

We defined LD domains of putatively functionally related elements, using a one-dimensional dynamic programming algorithm based on the mutual information (MI) content of all pairs of markers. A similar method was used previously with *D′* as the basis of comparison [35,38]. The treatment below can be equivalently implemented with *D′* replacing MI. The mutual information is defined by:


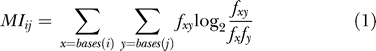


where the *x* and *y* summations are over all possible bases at markers *i* and *j,* respectively. The terms *f_x_* and *f_y_* are the observed frequencies of the indicated base at markers *i* and *j,* respectively, and *f_xy_* is the observed joint frequency for simultaneous occurrence of base *x* at marker *i* and base *y* at marker *j*.

MI varies between 0 and 1, but for dynamic programming to be stable, the average value of the sum term must be negative, therefore we used an offset of the mutual information, as defined by:


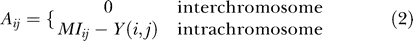



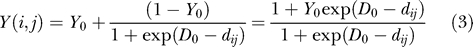


As shown, the term *A_ij_* is set identically to zero for marker pairs that are not on the same chromosome, since our domains are defined by proximity on a single chromosome. The offset *Y*(*i,j*) is dependent on markers *i* and *j* only through a sigmoid function of the distance separating the markers, *d_ij_*. At large separations, the offset function asymptotically approaches a value of 1.0, making all such marker pairs noncontributing to a domain.

*Y*(*i,j*) has two free parameters: *Y_0_,* the minimum value of the offset, and *D_0_,* the center of the sigmoid function. In practice, *Y_0_* was set as a quantile value (labeled as *q*) of the distribution of observed mutual information values for all interchromosomal pairs of markers. We typically set *q* between 0.95 and 0.999, however, other values were tested, as described below. The center of the sigmoid was typically set between 3 and 10 Mb (in practice, both *D_0_* and *d_ij_* were expressed in megabases), based on our observation (see Figure 1) that intrachromosomal markers become indistinguishable from interchromosomal markers, which necessarily cannot be part of a domain, for separations greater than 15–20 Mb.

All possible domain definitions were assessed by computing the triangular sum of all pairwise marker terms implied by the bounding markers *i* and *j,* again identically setting the term *S_ij_* to 0 if the bounding markers were not on the same chromosome:


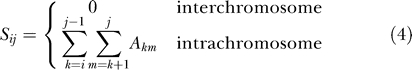


Finally, the optimal local domain structure was obtained in a one-dimensional Smith-Waterman [43] dynamic programming, based on the recursion relationship:


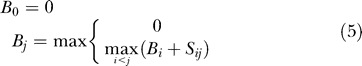


Local traceback (with subsequent reconstruction of the *B*-vector) was performed in decreasing order on all peak values greater than zero until all domains of at minimum two markers were identified.

To assess the robustness of the LD domain definitions, we implemented the algorithm for all combinations of *D_0_* = 1, 3, 5, 10, and 20 Mb, and *q* = 0.85, 0.9, 0.95, 0.99, 0.995, and 0.999, generating 30 different versions of the domains.

### Testing the effect of strain origins by rank-order test.

Groups 1 and 2 (Figure 1) are the two major groups of strains derived from domesticated mice; Group 3 includes wild-derived strains not influenced by domestication. The branch containing C57BL/6J was omitted to avoid marker bias, as this was the primary comparison strain for marker development. If the LD observed between marker pairs resulted from commonality of strain origins, there should be little similarity between the identities of the gene pairs in LD in the various groups.

The resulting group sizes are 22, 16, and 14, which greatly reduces the statistical power of a *D′*/FET analysis. To overcome this limitation, the marker pairs in each group were put in rank order on the basis of their calculated *p_FET_* values for LD, and the sum of the three rank orders was calculated. To assure comparability, this analysis was restricted to the 1,031 markers that are polymorphic within every group of Figure 1. If the LD among marker pairs is unrelated in the three groups, the sum of rank orders should be distributed as the sum of three integers that are randomly distributed between 1 and 530,965, the number of marker pairs.

## Abbreviations

FET: Fisher's exact test
GO: Gene Ontology
LD: linkage disequilibrium
RI: recombinant inbred
SNP: single-nucleotide polymorphism
